# Breast cancer and synchronous multiple primary lung adenocarcinomas with heterogeneous mutations: a case report

**DOI:** 10.1186/s12885-018-5011-4

**Published:** 2018-11-20

**Authors:** Bo Jin, Simeng Zhang, Xin Chuang, Ping Yu, Ying Chen, Yuee Teng, Xiaofang Che, Yibo Fan, Chunlei Zheng, Xiaohan Li, Xueshan Qiu, Xiujuan Qu, Yunpeng Liu

**Affiliations:** 1 Department of Medical Oncology, The First Hospital, China Medical University, Shenyang, China; 2 Department of Pathology, The First Hospital, China Medical University, Shenyang, China; 3 Department of pathology, Shengjing Hospital, China Medical University, Shenyang, China

**Keywords:** Multiple primary malignant tumors, EGFR mutation, Multiple primary lung adenocarcinomas, Synchronous

## Abstract

**Background:**

Multiple primary malignant tumors (MPMT) refers to the presence of two or more primary cancers of different organs in the same patient. MPMT is a sparse disease in the past, but there has been a gradual increase in the morbidity. Since multiple primary malignant tumors treatment methods differ, it is essential for clinicians to be able to distinguish between separate primary lesions and metastasis.

**Case presentation:**

We present the case of a 57-year-old woman with MPMT presenting with cancer in the left breast and synchronous double primary lung adenocarcinomas. We used IHC and epidermal growth factor receptor(EGFR)mutation to analyze genomic alteration profiles in the patient to validate the difference among the pathological assessments and the clinical differences between double primary lesions of lung and breast. EGFR gene analysis of breast cancer lesion revealed no mutations. The left and right lower lobe lung adenocarcinomas contained EGFR gene mutations: an L858R point mutation in exon 21 in the left lesion and a deletion mutation in exon 19 in the right lesion. The breast cancer and both lung adenocarcinomas were surgically resected. To date, the patient has remained disease-free.

**Conclusions:**

Both pathological and molecular assessment adapted in the current study appeared necessary. Mutational analysis of the EGFR gene provided important information not only in the diagnosis and but also in the treatment of MPMT.

## Background

Multiple primary malignant tumors (MPMT) refers to the presence of two or more primary cancers of different origins in the same patient. This uncommon condition is classified as synchronous or metachronous depending on the time of diagnosis: synchronous when the time between diagnosis of the first and second primary tumors is less than 6 months and metachronous when that period is more than 6 months [1]. The reported incidence of MPMT in cancer patients ranges from 0.73 to 11.7% among cancer patients [2, 3].

Here, we present a case of MPMT which have breast cancer and lung adenocarcinoma with heterogeneous epidermal growth factor receptor (EGFR) mutation status.

## Case presentation

**Fig. 1:**
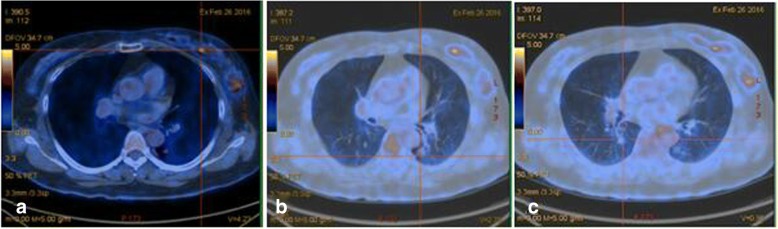
Positron Emission Tomography-computed tomography. The primary lesion in the left breast (**a**); The primary lesion in the left lower lobe of the lung (**b**) and the lesion in the right lower lobe of the lung (**c**)

In February 2016, a 57-year-old woman was admitted to our hospital for evaluation of a breast mass and multiple pulmonary nodules. AF^18^-fluorodeoxyglucose (FDG) positron emission tomography computed tomography (PET-CT) scan performed at the Shengjing Hospital of China Medical University showed a left breast mass with a FDG maximal standardized uptake value (SUVmax) of 4.23 (Fig. 1a), a left lower lung lobe (LLL) nodule measuring about 1.1 cm in diameter with increased FDG uptake (SUVmax = 2.79; Fig. 1b), and a right lower lung lobe (RLL) nodule measuring about 0.8 cm with normal FDG uptake (Fig. 1c). The LLL lesion was considered malignant, whereas the RLL lesion was not diagnosed as benign or malignant. Sequential surgery for resection of the breast cancer and LLL lesion was considered a reasonable course of action.

**Fig. 2:**
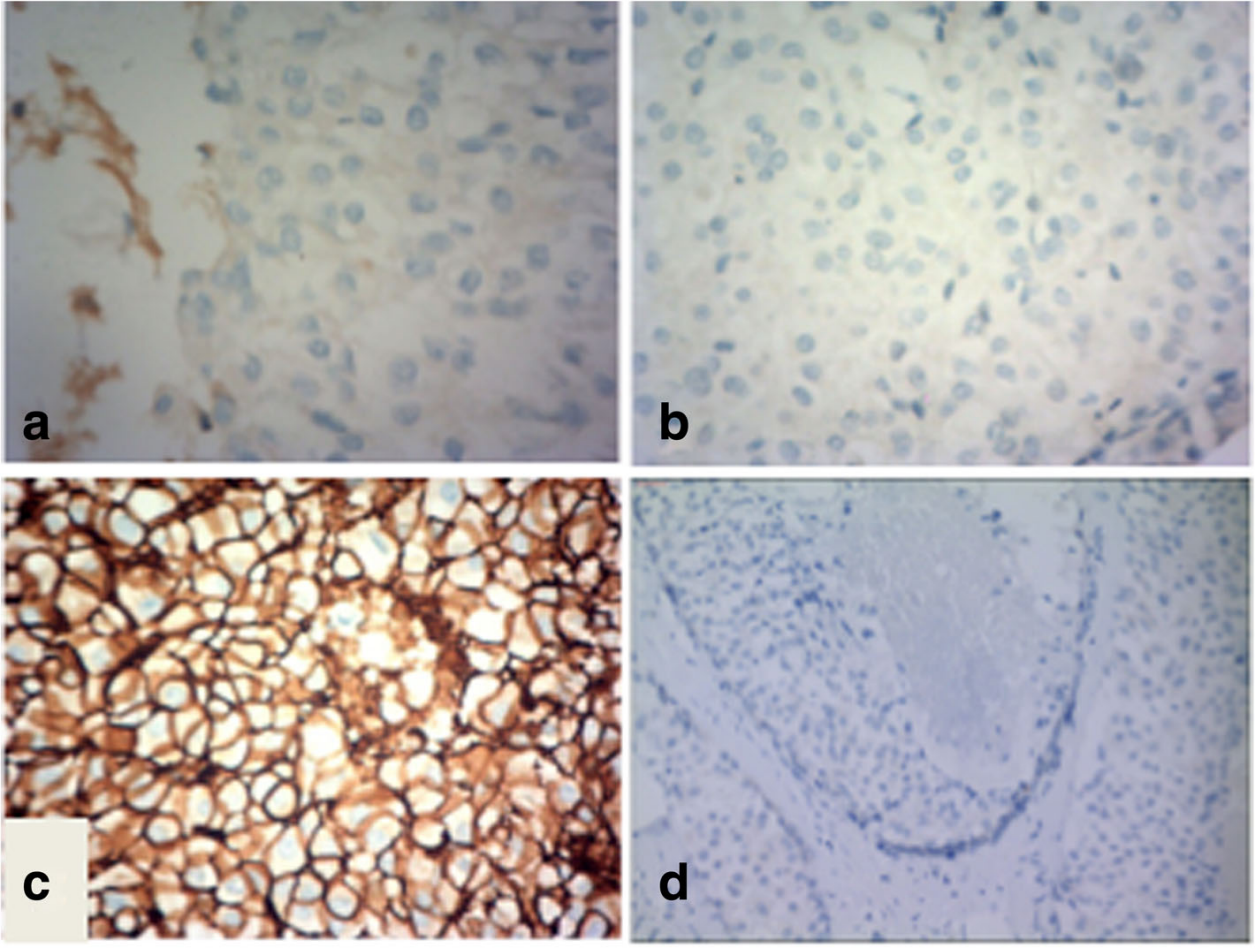
Immunohistopathological analysis of the postoperative breast lesion demonstrated negative staining for ER (**a**), PR (**b**) and TTF-1 (**d**), but positive staining for C-erbB-2 (**c**)

**Fig. 3:**
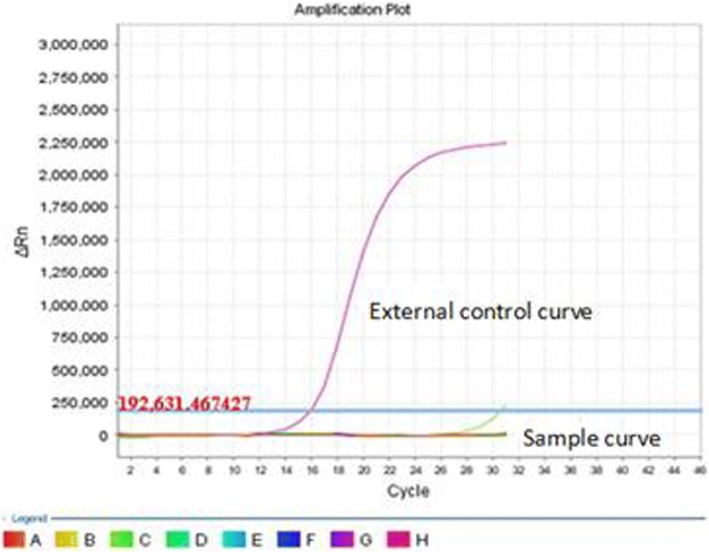
EGFR gene analysis of the left breast lesion revealed no mutations

A left radical mastectomy was performed on March 2nd, 2016. Postoperative pathology showed ductal carcinoma in situ (high grade). Immunohistochemical (IHC) staining indicated that the lesion was estrogen receptor(ER) negative (Fig. 2a), progesterone receptor(PR) negative (Fig. 2b), C-erbB-2 positive carcinoma in situ (3 +; Fig. 2c) and thyroid transcription factor-1(TTF-1) negative(Fig. 2d). The margins were negative. Sentinel lymph node analysis revealed reactive hyperplasia in the axillary lymph node (0/5,0/10). The pathological stage was pTisN0M0, 0 stage according to AJCC version 7.0 [4]. EGFR gene analysis (Fig. 3) revealed no mutations.

**Fig. 4:**
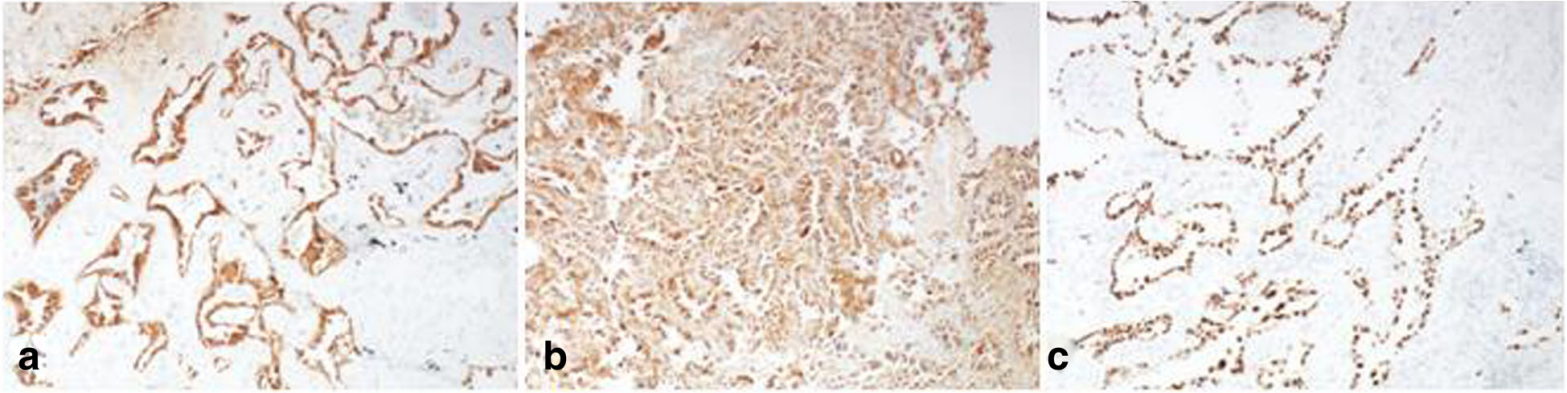
Immunohistopathological analysis of the postoperative left lung lesion demonstrated positive staining for CK7 (**a**), napsin A (**b**), and TTF-1 (**c**)

**Fig. 5:**
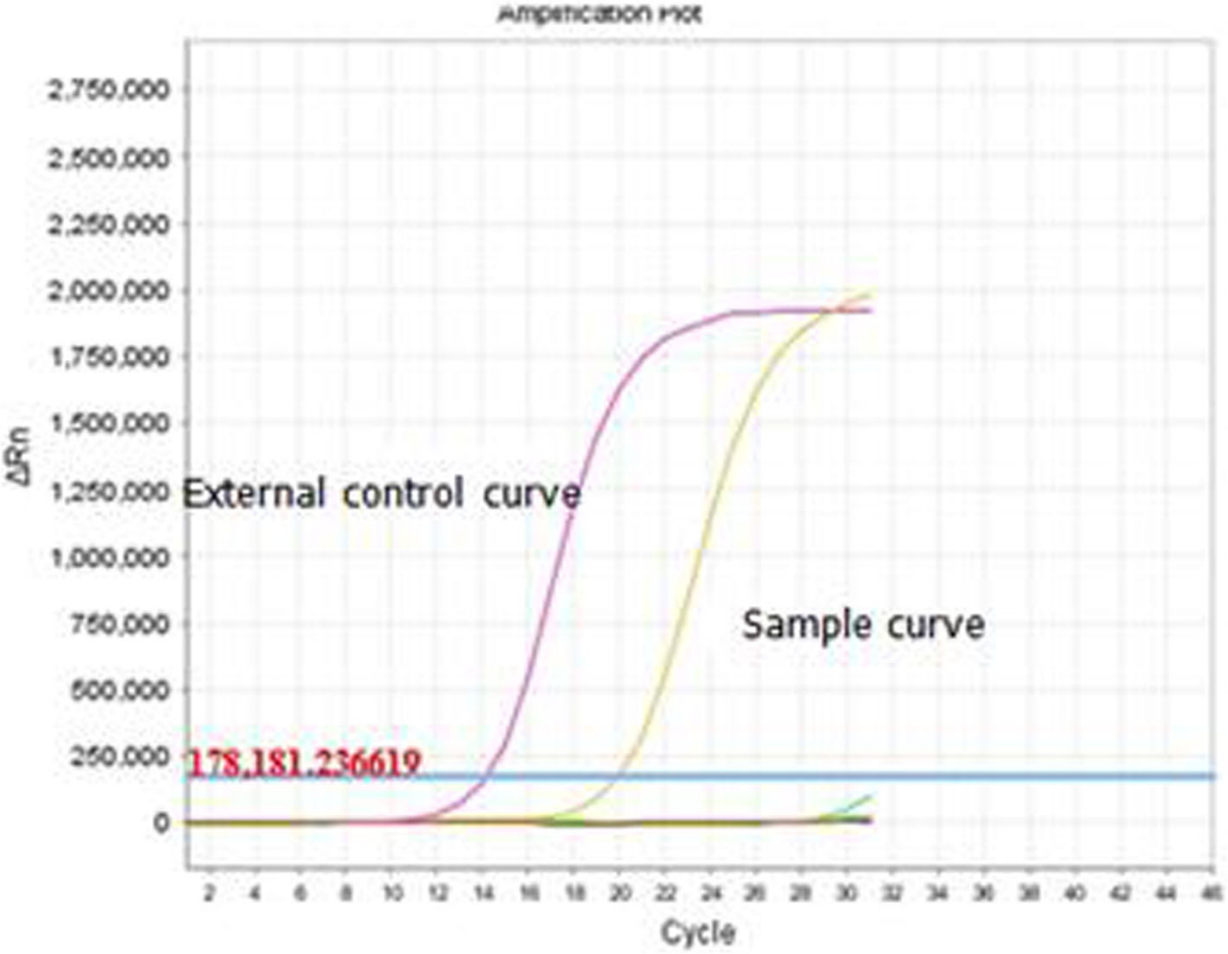
EGFR gene analysis of the left lung lesion revealed an L858R mutation in exon 21

A left lower lobectomy with lymph node dissection was performed at our hospital in April 2016. Postoperative pathology identified a highly to moderately differentiated adenocarcinoma (gland bubble type, 90%; lepidic growth pattern, 10%). Cancer cells were not detected in the lymph nodes. By IHC analysis, the lesion was CK7 positive (Fig. 4a), P63 negative, napsin A positive (Fig. 4b), TTF-1 positive (Fig. 4c), ALK D5F3 negative, ALK negative, and Ki-67 positive (5%). EGFR gene analysis was performed in June 2016 and showed an L858R mutation in exon 21 (Fig. 5). The pathological results of breast cancer (pTisN0M0,stage 0) and lung cancer (pT1aN0M0, stage IA according to AJCC version 7.0 [5]) showed that the patient has MPMT.

A chest CT scan performed 2 months after the left lower lobectomy showed that the small ground glass nodule in the RLL had increased in size compared with the scan performed 5 months earlier. Since the LLL lesion expressed mutated EGFR, the patient was prescribed gefitinib 250 mg daily starting in August 2016. A follow-up chest CT scan performed 40 days later showed a stable RLL nodule.

**Fig. 6:**
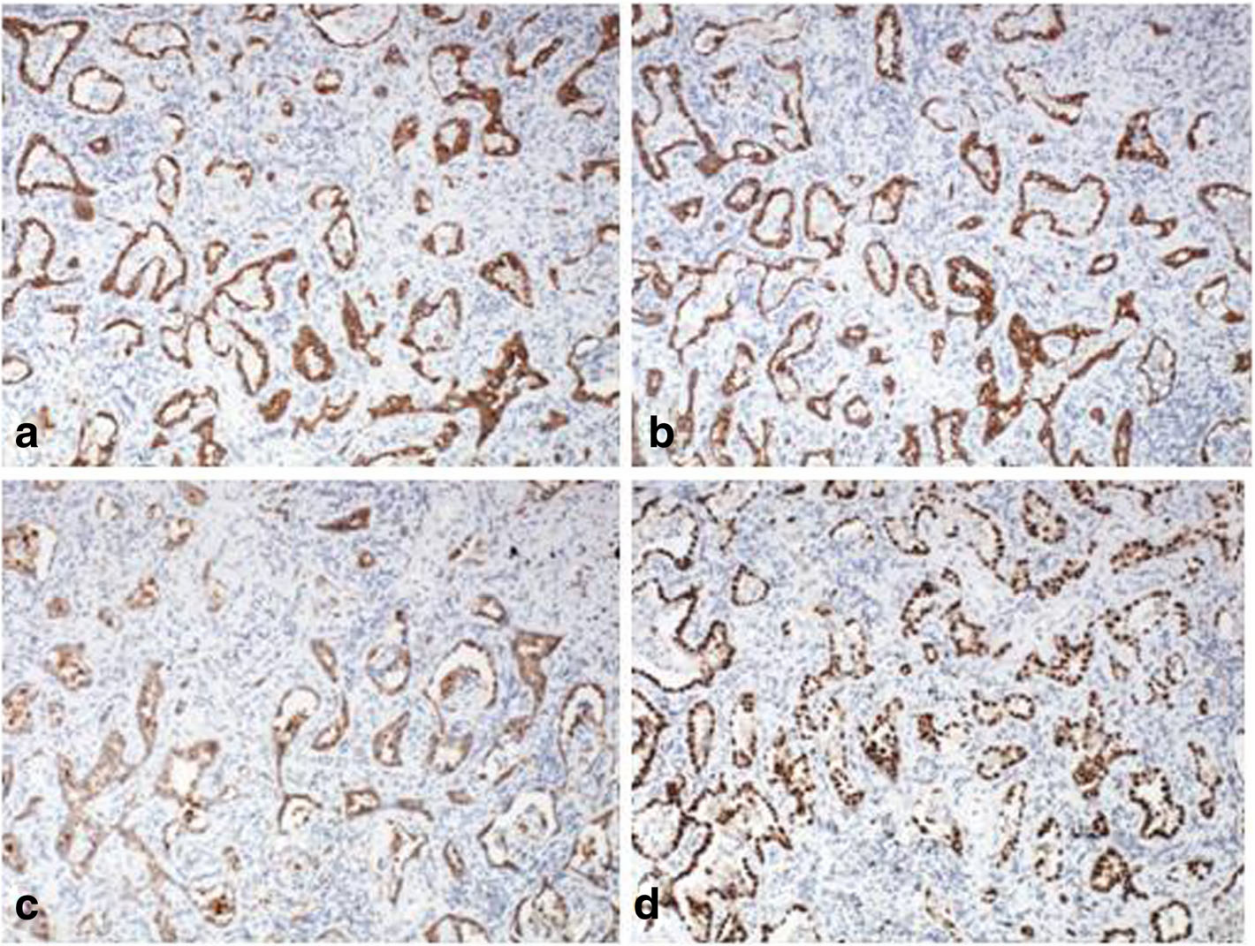
Immunohistopathological analysis of the postoperative right lung lesion demonstrated positive staining for CK A1 (**a**), CK7 (**b**), napsin A (**c**), and TTF-1 (**d**)

**Fig. 7:**
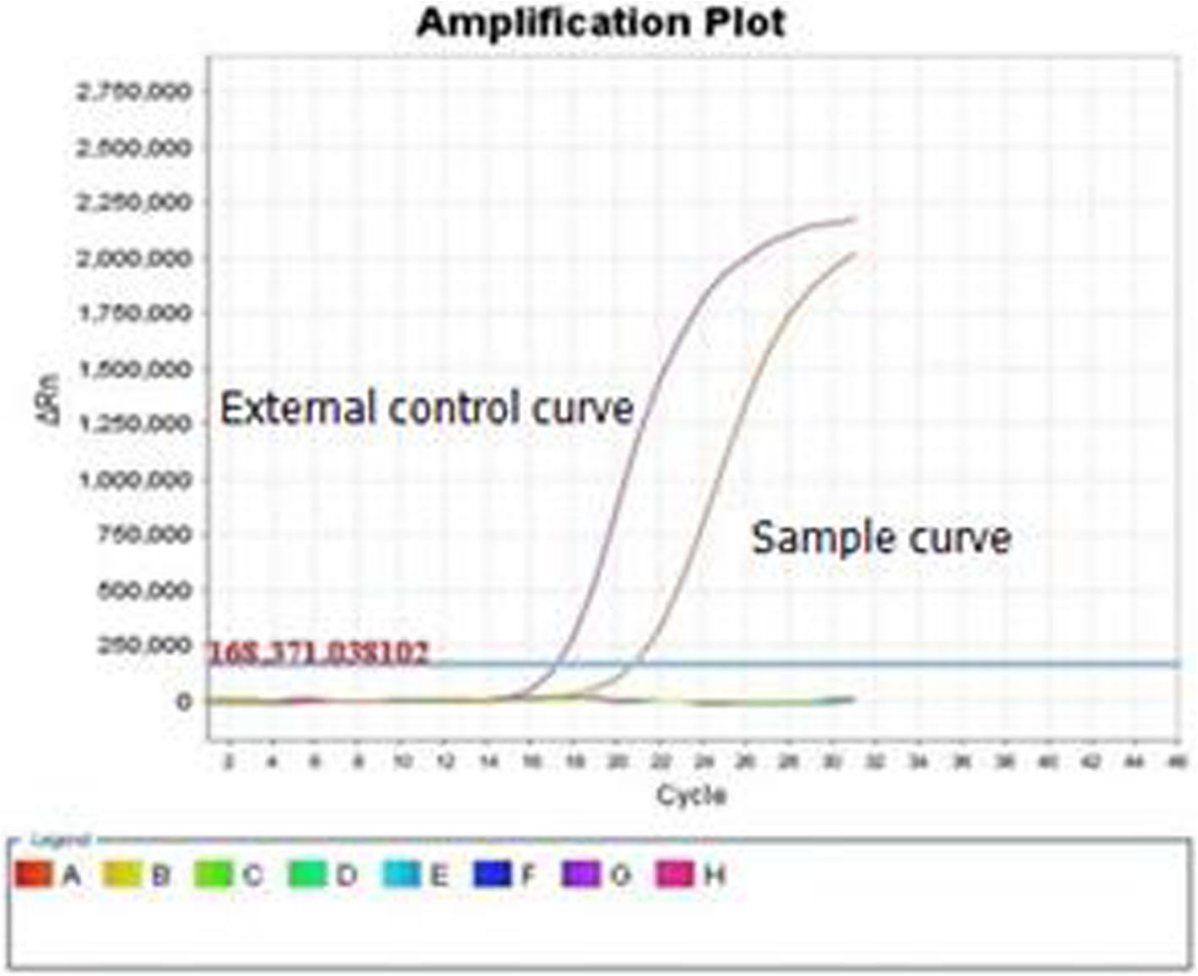
EGFR gene analysis of the right lung lesion revealed a deletion in exon 19

A right lower lobectomy with lymph node dissection was performed in our hospital in September 2016. Postoperative pathology identified an adenocarcinoma (alveolar type, 40%; growing along the alveolar wall, 60%). There was no lymph node metastasis and the surgical margins were clear. By IHC analysis, the lesion was CK A1 positive (Fig. 6a), CK5/6 negative, CK7 positive (Fig. 6b), P63 negative, P40 negative, napsin A positive (Fig. 6c), TTF-1 positive (Fig. 6d), CD56 negative, synaptophysin negative, and Ki-67 positive (10%). Analysis of the EGFR gene showed a deletion in exon 19 (Fig. 7). The pathological TNM stage was pT1aN0M0, stage IA according to AJCC version 7.0 [5].

## Discussion and conclusions

MPMT occur rarely, although consistent with the increased availability of diagnostic techniques, the number of patients diagnosed with MPMT is increasing. The diagnosis of MPLC can be difficult when multiple lung tumors of the same histologic type are present; similarly, it is not always easy to distinguish synchronous primary lesions from metastases. Analysis of gene mutations can be helpful in obtaining an accurate diagnosis. [6]

In the present case, the LLL lesion diagnosed concurrently with the breast cancer was also considered malignant, whereas the RLL lesion was not diagnosed as benign or malignant. We decided to surgically resect the breast cancer firstly. After that, we considered the LLL lesion was probably primary lung cancer instead of metastases from breast cancer because of the situ diagnosis in breast lesion. The pathological results of LLL showed that TTF-1 and Napsin A are positive and L858R mutation is detected in EGFR exon 21 which supported the diagnosis of primary lung cancer. To further identify the diagnosis of MPMT, we analyzed the EGFR mutation and TTF-1 status in breast lesion and found they were both negative. TTF-1 has been wildly used for differential diagnosis from lung adenocarcinoma with others and can be a useful marker in identifying lung tissue as a primary origin of metastases [7–10]. These results confirmed the diagnosis of MPMT.

The pathological stage of breast cancer and lung cancer suggested that there is no need for adjuvant therapy according to NCCN guideline. But, interestingly, the RLL lesion which firstly presented as small ground glass density nodules had been gradually increased in size in 6 months after initial diagnosis. This phenomenon highly indicated the malignant behavior. However, it was unclear whether the RLL lesion was a primary lung cancer or a metastasis from the breast or LLL lung cancers. Since the RLL lesion remained stable after administration of gefitinib for 40 days, and the patient’s performance status was suitable for surgery, we resected the RLL lesion. Pathological analysis confirmed that both the LLL and RLL lesions were adenocarcinoma of the lung with similar morphological features but with different EGFR mutations. The clinical and radiological presentation of the RLL lesion was indicative of a ground-glass opacity, and the pathological analysis confirmed a non-invasive adenocarcinoma. The sensitivity of the tumor to gefitinib treatment was consistent with the presence of EGFR mutation. The RLL and LLL lesions were considered to be of distinct origin because they contained different EGFR mutations.

The final diagnosis in the present case was breast cancer with synchronous primary lung cancers. Given the different mutational status of the EGFR gene in the two lung adenocarcinomas, it is reasonable to assume that they are oncodevelopmentally independent. The patient remains disease-free as of Aug 16th 2018.

## Abbreviations

EGFR: Epidermal growth factor receptor
ER: Estrogen receptor
FDG: F^18^-fluorodeoxyglucose
IHC: Immunohistochemical
LLL: Lower lung lobe
MPLC: Multiple primary lung cancer
MPMT: Multiple primary malignant tumors
NSCLC: Non small cell lung cancer
PET-CT: Positron emission tomography-computed tomography
PR: Progesterone receptor
RLL: Right lower lung lobe
SUV: Standardized uptake value
TNM: Tumor Lymph node Metastasis stage
